# Evidence of Vent-Adaptation in Sponges Living at the Periphery of Hydrothermal Vent Environments: Ecological and Evolutionary Implications

**DOI:** 10.3389/fmicb.2020.01636

**Published:** 2020-07-24

**Authors:** Magdalena N. Georgieva, Sergi Taboada, Ana Riesgo, Cristina Díez-Vives, Fabio C. De Leo, Rachel M. Jeffreys, Jonathan T. Copley, Crispin T. S. Little, Pilar Ríos, Javier Cristobo, Jon T. Hestetun, Adrian G. Glover

**Affiliations:** ^1^Life Sciences Department, Natural History Museum, London, United Kingdom; ^2^Departamento de Biología (Zoología), Universidad Autónoma de Madrid, Madrid, Spain; ^3^Departamento de Zoología y Antropología Física, Universidad de Alcalá, Madrid, Spain; ^4^Ocean Networks Canada, University of Victoria, Victoria, BC, Canada; ^5^Department of Biology, University of Victoria, Victoria, BC, Canada; ^6^School of Environmental Sciences, University of Liverpool, Liverpool, United Kingdom; ^7^School of Ocean and Earth Science, University of Southampton, Southampton, United Kingdom; ^8^School of Earth and Environment, University of Leeds, Leeds, United Kingdom; ^9^Centro Oceanográfico de Santander, Instituto Español de Oceanografía, Santander, Spain; ^10^Centro Oceanográfico de Gijón, Instituto Español de Oceanografía, Gijón, Spain; ^11^NORCE Environment, Norwegian Research Centre (NORCE), Bergen, Norway

**Keywords:** Porifera, 16S rRNA amplicon, microbiome, nutrition, chemosynthesis, cold seep

## Abstract

The peripheral areas of deep-sea hydrothermal vents are often inhabited by an assemblage of animals distinct to those living close to vent chimneys. For many such taxa, it is considered that peak abundances in the vent periphery relate to the availability of hard substrate as well as the increased concentrations of organic matter generated at vents, compared to background areas. However, the peripheries of vents are less well-studied than the assemblages of vent-endemic taxa, and the mechanisms through which peripheral fauna may benefit from vent environments are generally unknown. Understanding this is crucial for evaluating the sphere of influence of hydrothermal vents and managing the impacts of future human activity within these environments, as well as offering insights into the processes of metazoan adaptation to vents. In this study, we explored the evolutionary histories, microbiomes and nutritional sources of two distantly-related sponge types living at the periphery of active hydrothermal vents in two different geological settings (*Cladorhiza* from the E2 vent site on the East Scotia Ridge, Southern Ocean, and *Spinularia* from the Endeavour vent site on the Juan de Fuca Ridge, North-East Pacific) to examine their relationship to nearby venting. Our results uncovered a close sister relationship between the majority of our E2 *Cladorhiza* specimens and the species *Cladorhiza methanophila*, known to harbor and obtain nutrition from methanotrophic symbionts at cold seeps. Our microbiome analyses demonstrated that both E2 *Cladorhiza* and Endeavour *Spinularia* sp. are associated with putative chemosynthetic Gammaproteobacteria, including Thioglobaceae (present in both sponge types) and Methylomonaceae (present in *Spinularia* sp.). These bacteria are closely related to chemoautotrophic symbionts of bathymodiolin mussels. Both vent-peripheral sponges demonstrate carbon and nitrogen isotopic signatures consistent with contributions to nutrition from chemosynthesis. This study expands the number of known associations between metazoans and potentially chemosynthetic Gammaproteobacteria, indicating that they can be incredibly widespread and also occur away from the immediate vicinity of chemosynthetic environments in the vent-periphery, where these sponges may be adapted to benefit from dispersed vent fluids.

## Introduction

Deep-sea hydrothermal vents are remarkable environments generally characterized by low diversity, high abundance communities (relative to other deep-sea environments at similar depths), supported by *in situ* chemosynthetic primary production using reduced substrates dissolved in vent fluid. Many vent-specialist metazoans show close symbiotic associations with chemosynthetic microbes, on which they are nutritionally dependent (Dubilier et al., 2008). Characteristic vent taxa such as siboglinid tubeworms, bathymodiolin mussels, and *Kiwa* anomuran crabs harbor microbial symbionts either internally or on their exterior surfaces, most commonly belonging to the bacterial classes Gammaproteobacteria and Epsilonproteobacteria (e.g., Fujiwara et al., 2000; Thornhill et al., 2008; Zwirglmaier et al., 2015). To ensure their symbionts have optimal access to vent fluid, many vent-endemic taxa live within a few meters of vent chimneys, demonstrating zonation of species structured by nutritional modes and temporal succession (e.g., Marsh et al., 2012).

In addition to taxa endemic to chemosynthetic environments, a suite of animals occurs more peripherally at vents, tens to hundreds of meters from active chimneys (Marcus and Tunnicliffe, 2002; Bernardino et al., 2012). These peripheral taxa often include suspension-feeders, scavengers, and predators that are known from non-chemosynthetic environments, but occur at increased abundance in a “halo” assemblage around vent fields. Filter-feeding taxa such as serpulid worms (ten Hove and Zibrowius, 1986) and sponges (Hestetun et al., 2017; Kelly and Rowden, 2019) at the vent periphery are inferred to benefit from the availability of hard substrate and increased amounts of food suspended in the water column. However, for many taxa whose increased abundance at the periphery of vents indicates that they benefit from the vent environment, it is not known specifically how they benefit. This knowledge is essential for assessing the vent sphere of influence, which has implications for the management of human activities within these environments, as well as for understanding how animals may become more intimately adapted to hydrothermal vents.

Sponges, which most commonly feed through filtration but can also be carnivorous (Vacelet and Boury-Esnault, 1995; Vacelet, 2007), are known to associate with stable and phylogenetically diverse microbial communities, that contribute to key functions such as metabolism (Taylor et al., 2007; Webster and Blackall, 2009). Sponges are also often found living on the periphery or amongst specialists in chemosynthetic habitats such as hydrothermal vents and cold seeps (e.g., Boury-Esnault and De Vos, 1988; Olu-Le Roy et al., 2004; Vacelet, 2006; Schander et al., 2010; Collins et al., 2012). Within the above environments, sponges from across the phylum Porifera are known to harbor high abundances of sulfide and methane-oxidizing bacteria as part of their microbiomes, indicating that some sponges can also be nutritionally reliant on chemosynthetic symbionts (Vacelet et al., 1996; Nishijima et al., 2010; Petersen et al., 2012; Arellano et al., 2013; Zhou et al., 2019). Recently, two distantly-related sponges from asphalt seeps in the Gulf of Mexico [*Hymedesmia* (*Stylopus*) *methanophila* and *Iophon methanophila*] were found to obtain their nutrition primarily from methane-oxidizing endosymbionts (Rubin-Blum et al., 2019). The sponge species *Cladorhiza methanophila*, which belongs to the carnivorous family Cladorhizidae, also hosts methanotrophic symbionts from which it is inferred to feed (Vacelet et al., 1995; Hestetun et al., 2016a). The microbes shown to be important in these associations often belong to gammaproteobacterial lineages such as Methylococcales (Hestetun et al., 2016a), Marine Methylotrophic Group 2 (Rubin-Blum et al., 2019), *Methylohalomonas* (Arellano et al., 2013), and the SUP05 clade (Zhou et al., 2019). Such convergence in symbiont acquisition likely confers sponges a strong selective advantage in the generally food-limited deep sea. While some sponges appear to have become more intimately adapted to a chemosynthetic mode of life, for other vent-peripheral and non-vent sponges chemolithotrophy (the acquisition of energy from the oxidation of inorganic compounds) appears to be a means through which sponges might supplement their nutrition in the deep ocean (Kennedy et al., 2014; Hestetun et al., 2016a; Tian et al., 2016). For deep-sea sponges that do not occur in chemosynthetic environments, microbial groups such as ammonia-oxidizing Thaumarchaeota archaea also appear to be important symbionts in addition to the Gammaproteobacteria (Kennedy et al., 2014).

Carnivorous cladorhizid sponges are particularly abundant at the periphery of hydrothermal vents. At the E2 site, East Scotia Ridge, Southern Ocean, individuals of *Cladorhiza* occur beyond the area of vent-specialist taxa (Marsh, 2014), in a concentric zone around the vent chimneys (L. Marsh, *pers. comm.*). At the Endeavour hydrothermal vent site, Juan de Fuca Ridge, North-East Pacific, there are few conspicuous vent peripheral animals although a number of mostly filter-feeding sponges have also been observed in proximity to the Endeavour vents (Ocean-Networks-Canada, 2017). The microbial associates of these vent-peripheral sponges are unknown, and analysis of the composition of their microbiomes has the potential to shed insights into the nature of interactions between sponges and nearby chemosynthetic environments (Hestetun et al., 2016a; Zhou et al., 2019). Analyses of stable isotopes of carbon (^13^C), nitrogen (^15^N), and sulfur (^34^S) have also been employed to assess contributions of chemosynthetic sources of nutrition to sponges occurring at hydrothermal vent and cold seep sites, whereby depletions in the above isotopes generally point toward a greater influence of chemosynthesis to sponge diets (Erickson et al., 2009; Reid et al., 2013; Hestetun et al., 2016a).

This study aims to test the hypothesis that, independent of feeding mode, sponges have adapted to a peripheral vent habitat through association with chemoautotrophic microbes. We used a combination of molecular tools and stable isotope analysis to test this hypothesis. Phylogenetic analyses were used to assess relatedness to known sponges with chemosynthetic microbial symbionts, sponge microbiomes were examined to determine if the sponges were associating with vent-specific microbes, and finally stable isotope measurements were used to explore sponge nutritional sources.

## Materials and Methods

### Sampling

**FIGURE 1:**
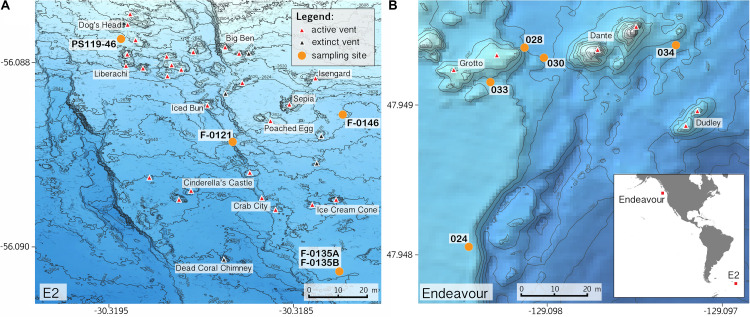
Locations of sponge sampling around vent chimneys at **(A)** E2, East Scotia Ridge, and at **(B)** Endeavour, Juan de Fuca Ridge, source of bathymetry data: Kelley et al. (2015). Inset map in B shows location of the two vent sites. Note: position of sample PS119-46 is approximate.

**FIGURE 2:**
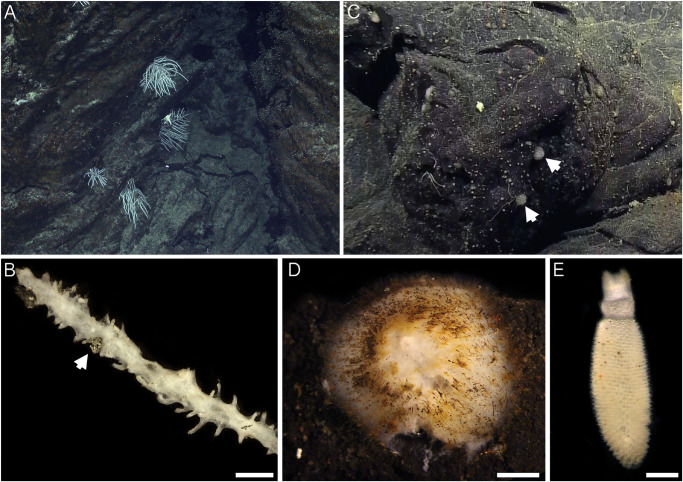
Sponges from E2 and Endeavour sites analyzed during the present study. **(A)** *Cladorhiza in situ* at E2. **(B)** detail of E2 *Cladorhiza* branch (specimen PS119-046), arrow shows pyrite grain among the branches. Scale bar is 2 mm. **(C)** *Spinularia* (arrowed) *in situ* at Endeavour shortly before collection. **(D)** Detail of Endeavour *Spinularia* (specimen 028-S2.2), scale bar is 2 mm. **(E)** Detail of *Sycon* sponge collected alongside Endeavour *Spinularia* specimens, scale bar is 1 mm.

Sample collections of vent-peripheral sponges (Figure 1) took place on three expeditions to deep-sea hydrothermal vents. *Cladorhiza* (Figures 2A,B; Porifera: Cladorhizidae) specimens were collected from the E2 vent field on the East Scotia Ridge, Southern Ocean (Rogers et al., 2012) during RRS *James Cook* JC042 (January–February 2010) and RV *Polarstern* PS119 (April–May 2019; Bohrmann, 2019). *Spinularia* and *Sycon* (Figures 2C–E; Porifera: Polymastiidae and Sycettidae, respectively) specimens were collected during a July 2018 voyage to the Endeavour hydrothermal vent site, Juan de Fuca Ridge, by RV *Nautilus* NA098 (Table 1). The majority of sponge specimens were collected by remotely operated vehicles (ROVs) within approximately 20 m of active hydrothermal vents (Figure 1), with the closest specimens located less than 10 m from vent chimneys. JC042 specimens were preserved in 80% ethanol, and PS119 and NA098 specimens were preserved in RNAlater. RNAlater specimens were kept at −80°C following collection and during subsequent long-term storage. In addition, non-vent *Chondrocladia* specimens were collected during expeditions to the Norwegian Sea (Mareano expedition 113, RV *G. O. Sars*, October 2011), the Aviles Canyon System in the Cantabrian Sea off northern Spain (SponGES0617 expedition, RV *Angeles Alvariño*, June 2017), offshore eastern Patagonia (Patagonia 1208 expedition, RV *Miguel Oliver*, December 2008), and the eastern Gulf of Mexico (expedition EX1711, NOAAS *Okeanos Explorer*, December 2017) (Table 1) and used for microbiome comparisons with E2 *Cladorhiza* specimens.

**TABLE 1:** Specimens analyzed in the present study.

Expedition	ROV dive	Taxon	Sample code	Tissue type	Preservation	Vent site	Latitude	Longitude	Depth (m)	Analyses
***Dataset 1: Cladorhiza-Chondrocladia***										
JC042	ISIS133	*Cladorhiza* sp.	F-0121 (MG97)	stalk+branches	80% ethanol	E2, East Scotia Ridge	−56.088930	−30.318330	2584	mb
JC042	ISIS134	*Cladorhiza* sp.	F-0135A (MG98)	stalk+branches	80% ethanol	E2, East Scotia Ridge	−56.089633	−30.317750	2582	phy, mb
JC042	ISIS134	*Cladorhiza* sp.	F-0135A (M2)	stalk	80% ethanol	E2, East Scotia Ridge	−56.089633	−30.317750	2582	phy, mb
JC042	ISIS134	*Cladorhiza* sp.	F-0135B (MG70)	stalk	80% ethanol	E2, East Scotia Ridge	−56.089633	−30.317750	2582	phy, mb
JC042	ISIS134	*Cladorhiza* sp.	F-0135B (MG69)	branches	80% ethanol	E2, East Scotia Ridge	−56.089633	−30.317750	2582	phy, mb
JC042	ISIS134	*Cladorhiza* sp.	F-0146 (MG71)	stalk	80% ethanol	E2, East Scotia Ridge	−56.088783	−30.317733	2582	phy
PS119	QUEST446	*Cladorhiza* sp.	PS119-46	stalk+branches	RNAlater	E2, East Scotia Ridge	−56.088798	−30.319186	2641	phy, iso
Mareano113	R818	*Chondrocladia grandis*	2012	stalk	ethanol	Norway	67.59933	−9.31983	913	mb
Mareano113	R818	*Chondrocladia grandis*	2012	sphere	ethanol	Norway	67.59933	−9.31983	913	mb
SponGES0617	BT6	*Chondrocladia robertballardi*	BT6602C	stalk	96% ethanol	Cantabrian Sea	43.9811	−6.47703	1525	mb
SponGES0617	BT6	*Chondrocladia robertballardi*	BT6602C	sphere	96% ethanol	Cantabrian Sea	43.9811	−6.47703	1525	mb
PATAGONIA1208	46DR5	*Chondrocladia* sp.	PATAGONIA46	stalk	70% ethanol	Offshore eastern Patagonia	−45.654833	−59.664483	1320	mb
PATAGONIA1208	46DR5	*Chondrocladia* sp.	PATAGONIA46	sphere	70% ethanol	Offshore eastern Patagonia	−45.654833	−59.664483	1320	mb
EX1711	DIVE01	*Chondrocladia verticillata*	USNM1482939	stalk	ethanol	Gulf of Mexico	24.65	−83.91	735	mb
EX1711	DIVE01	*Chondrocladia verticillata*	USNM1482939	sphere	ethanol	Gulf of Mexico	24.65	−83.91	735	mb
***Dataset 2: Spinularia-Sycon***										
NA098	H1695	*Spinularia* sp.	024-S1	−	RNAlater	Endeavour, JdF Ridge	47.948410	−129.098518	2194	phy, mb
NA098	H1695	*Spinularia* sp.	028-S2.1	−	RNAlater	Endeavour, JdF Ridge	47.949220	−129.098281	2192	phy, mb, iso
NA098	H1695	*Spinularia* sp.	028-S2.2	−	RNAlater	Endeavour, JdF Ridge	47.949220	−129.098281	2192	phy, mb
NA098	H1695	*Spinularia* sp.	028-S2.3	−	RNAlater	Endeavour, JdF Ridge	47.949220	−129.098281	2192	phy, mb
NA098	H1695	*Spinularia* sp.	030-S4	−	RNAlater	Endeavour, JdF Ridge	47.949191	−129.098213	2194	phy, mb
NA098	H1696	*Spinularia* sp.	033-S1.1	−	RNAlater	Endeavour, JdF Ridge	47.949092	−129.098421	2194	phy, mb
NA098	H1696	*Spinularia* sp.	033-S1.2	−	RNAlater	Endeavour, JdF Ridge	47.949092	−129.098421	2194	phy, mb
NA098	H1696	*Spinularia* sp.	034-S2.1	−	RNAlater	Endeavour, JdF Ridge	47.949242	−129.097689	2198	phy, mb
NA098	H1696	*Spinularia* sp.	034-S2.2	−	RNAlater	Endeavour, JdF Ridge	47.949242	−129.097689	2198	phy, mb
NA098	H1696	*Spinularia* sp.	034-S2.3	−	RNAlater	Endeavour, JdF Ridge	47.949242	−129.097689	2198	phy, mb, iso
NA098	H1696	*Spinularia* sp.	034-S2.4	−	RNAlater	Endeavour, JdF Ridge	47.949242	−129.097689	2198	phy, mb
NA098	H1696	*Sycon* sp.	033-S1.3	−	RNAlater	Endeavour, JdF Ridge	47.949092	−129.098421	2194	mb

*mb, microbiomes; phy, phylogenetics; iso, isotopic analysis; JdF, Juan de Fuca.*

### DNA Extraction, Amplification and Sequencing of Host Genes

Sponge tissues were rinsed in ethanol, finely-chopped, and DNA was subsequently extracted from approximately 6 mm long specimen fragments using either an E.Z.N.A. Soil DNA Kit (*Chondrocladia* specimens) or a Qiagen DNeasy Blood & Tissue Kit (*Cladorhiza* and *Spinularia* specimens), following instructions provided by the manufacturer with the modification of final elution in 100 μl. A subset of DNA extractions were performed on different sponge tissue types for the microbiomes work (see section “Microbiome Amplification and Sequencing”).

For *Cladorhiza* specimens, the overlapping “Folmer” and “Erpenbeck” fragments of cytochrome c oxidase subunit I (COI; 500–1100 bp), the C1-D2 partition of 28S rRNA (28S; 340–780 bp), and part of the protein-coding gene alpha-1,2-Mannosyltransferase (ALG11; 640–790 bp) were amplified using primers and polymerase chain reaction (PCR) protocols outlined in Hestetun et al. (2016b) (Supplementary Table S1). All gene fragments for *Cladorhiza* and *Spinularia* specimens were amplified in 12.5 μl reactions, made up of 10.5 μl Red Taq DNA Polymerase 1.1X MasterMix (VWR), 0.5 μl of each primer, and 1 μl of DNA template. For unsuccessful PCRs, using 1.5 μl DNA template, and increasing or decreasing the annealing temperature depending on whether no bands or multiple bands were observed improved sequencing success. The ALG11 D2-R2 PCR program was modified as follows: (94°C/5 min, 54°C/2 min, 72°C/2 min)^∗^1 cycle, (94°C/1 min, 58°C/30 s, 72°C/1 min)^∗^35 cycles, 72°C/7 min. For *Spinularia* sp. specimens, COI (∼690 bp) and the D1-D2, D3-D5, and D6-D8 regions of 28S (650–840 bp) were amplified using primers outlined in Supplementary Table S1 and the following PCR protocol: 94°C/5 min, (94°C/45 s, 55°C/45 s, 72°C/2 min)^∗^35 cycles, 72°C/10 min. Following electrophoresis, PCR products were visualized on 1% agarose gels, purified and subsequently sequenced using an ABI 3730XL DNA Analyzer (Applied Biosystems) at the Natural History Museum Sequencing Facility, United Kingdom (NHM).

### Phylogenetic Analysis

The newly-generated sequences were aligned with existing Cladorhizidae (for the dataset containing E2 *Cladorhiza*) and Polymastiidae (for the dataset containing Endeavour *Spinularia*) sequences available on the National Center for Biotechnology Information (NCBI) GenBank (Supplementary Tables S2, S3) using Geneious v.10.2.5 (Kearse et al., 2012) and the MUSCLE (Edgar, 2004) alignment option available therein. For Cladorhizidae 28S, the program Gblocks v.0.91b (Castresana, 2000) was used to remove gaps and uncertain positions in the alignment. jModelTest v.2.1.10 (Guindon and Gascuel, 2003; Darriba et al., 2012) was used to select the best fitting model for each gene alignment according to the Akaike Information Criterion, which was GTR+I+G for Polymastiidae 28S and Cladorhizidae COI, GTR+G for Polymastiidae COI and Cladorhizidae 28S, and HKY+G for Cladorhizidae ALG11. Phylogenetic analyses were performed using MrBayes v.3.2.6 (Ronquist et al., 2012) on a concatenated dataset of COI, 28S, and ALG11 for Cladorhizidae, and COI concatenated with 28S for Polymastiidae. Each analysis was run for 10,000,000 generations (with 2,500,000 discarded as burn-in) using the above models. Convergence was checked using Tracer v1.7 (Rambaut et al., 2018). Genetic distances (uncorrected *p*-distance) amongst members of the genus *Cladorhiza* (using three datasets separately: COI, 28S, ALG11) and the family Polymastiidae (using two datasets separately: COI, 28S) were calculated using PAUP^∗^ v.4.0a (build 166; Swofford, 2002).

### Microbiome Amplification and Sequencing

As the type of tissue selected for DNA extraction can have an effect on the resulting microbiome in Cladorhizidae (Verhoeven et al., 2017), for our *Cladorhiza* and *Chondrocladia* specimens DNA extractions from either the stalk, branches/sphere, or stalk and branches/sphere combined (Table 1) were used to check for such effects in our samples.

The V4 variable region of 16S SSU rRNA gene was targeted for microbiome sequencing using the prokaryote primers 515F-Y (Parada et al., 2016) and 806R (Apprill et al., 2015). Mixtures for initial PCR amplification contained 0.25 μl of each primer, 1 μl DNA template, 8.875 μl ddH_2_0, 2.5 μl 5× PCRBIO HiFi buffer, and 0.125 μl PCRBIO HiFi Polymerase giving a total reaction volume of 13 μl. The PCR program was as follows: 95°C/5 min, (95°C/20 s, 60°C/20 s, 72°C/30 s)^∗^25 cycles, 72°C/5 min. DNA amplification was done in duplicates to mitigate PCR biases. Successful reactions (based on 1% agarose gel visualization) were purified using Agencourt AMPure XP beads (Beckman Coulter Inc., United States), and libraries were prepared using the NextEra XT DNA Library Preparation Kit (Illumina Inc., United States). Samples were subsequently pooled in equimolar concentrations (normalization of all samples to 4 nM), and sequenced on an Illumina MiSeq platform at the NHM in 2 × 300 bp mode (paired-end). The resulting amplicon sequence lengths were ca. 298 bp for the V4 region.

Microbiome sequencing was performed in two separate runs forming two datasets (Table 1 and Supplementary Table S4) that were not analyzed together to avoid between-run biases. The *Cladorhiza-Chondrocladia* dataset (Dataset 1) was comprised of E2 *Cladorhiza* vent-peripheral specimens and samples of closely-related *Chondrocladia* spp. from non-vent environments. For Endeavour *Spinularia* sp. specimens, non-vent samples of other polymastiids were not available for comparison, however the microbiome of an additional sponge (*Sycon* sp., Figure 2E) from the Endeavour site collected alongside *Spinularia* sp. was included in the analyses, thus forming the *Spinularia-Sycon* dataset (Dataset 2).

### Microbiome Data Processing

The software *mothur* (Schloss et al., 2009) was used to firstly join paired end reads, and remove primer sequences, ambiguous bases and homopolymers. After identification of unique sequences, these were subsequently aligned to the SILVA SEED v132 reference database (Quast et al., 2012). Amplicon sequence variants (ASV) were inferred allowing 1 mismatch per 100 nucleotides (Callahan et al., 2016), then checked for chimeras against the SILVA GOLD database, and classified taxonomically against the SILVA NR v132 database.

In R (R Core Team, 2013), ASV counts were converted to relative abundance and used to calculate Bray-Curtis dissimilarities, perform non-metric multidimensional scaling ordination analysis (nMDS), as well as to run permutational multivariate ANOVA (PERMANOVA) to examine whether variation in the microbiomes could be attributed to host taxonomy, body part, and sampling location factors. Relative abundances of ASVs were also used to compute weighted UniFrac distances (Lozupone and Knight, 2005) between the samples of each dataset using the R packages *ape* (Paradis et al., 2004) and *phyloseq* (McMurdie and Holmes, 2013). ASV relative abundances were also pooled into taxonomic units, and the data were analyzed using the R package *vegan* (Oksanen et al., 2010) and *ape*, and visualized using *ggplot2* (Wickham, 2011) and *pheatmap* (Kolde, 2015).

A published dataset of several Cladorhizidae sponge microbiomes from near vent and cold seep environments (Hestetun et al., 2016a), obtained using near-identical primers to those used in this study (519F-805R), was also re-analyzed here according to the above steps (forming Dataset 3) to permit a more direct comparison with our cladorhizid microbiome data.

### Isotopic Analysis

Stable isotope analyses of δ^13^C and δ^15^N were performed at the Liverpool Isotopes for Environmental Research Laboratory, University of Liverpool, United Kingdom. Isotope ratios are reported here in standard δ-notation (‰) relative to the Vienna Pee Dee Belemnite (δ^13^C) and atmospheric N_2_ (δ^15^N). Samples were analyzed using an elemental analyzer (Costech) coupled to a Delta V isotope ratio mass spectrometer (IRMS; Thermo-Scientific). To ensure accuracy, certified reference materials USGS40 and USGS41a were analyzed at the beginning, middle and end of each run and generated values that were within ≤0.2‰ of certified values for both δ^13^C and δ^15^N. An internal reference material of ground prawn (*Penaeus vannamei*) with well characterized isotope values (δ^13^C -22.6‰ and δ^15^N 6.8‰) was analyzed every 10 samples to monitor precision, which was 0.1‰ for both δ^13^C and δ15N. Prior to stable carbon isotope analyses, lipids were extracted from sponges using a modified method of Bligh and Dyer (1959). Briefly, sponges were weighed into a glass centrifuge tube, and 3 mL of 2:1 dichloromethane to methanol solution was added to each sponge and sonicated at room temperature for 30 min. After sonication, the organic solvents were removed and the process was repeated twice. After the final sonication, samples were frozen and freeze-dried. Dried sponges were then acidified following the methods of Yamamuro and Kayanne (1995). Stable nitrogen analyses were carried out on untreated samples.

## Results

### Sponge Phylogenies

**FIGURE 3:**
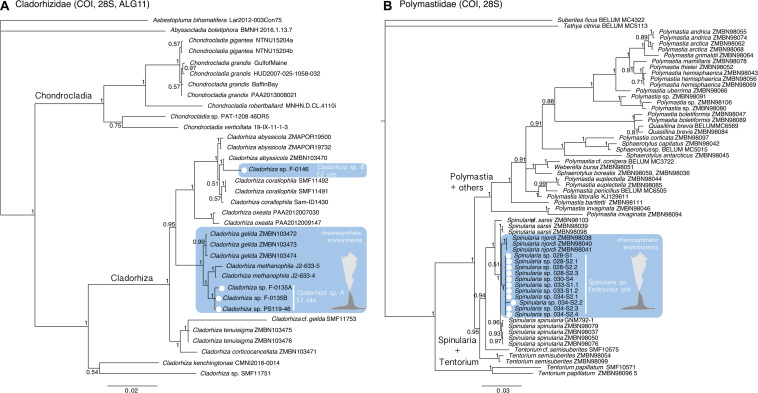
Bayesian phylogenetic analyses of **(A)** Cladorhizidae based on a concatenated dataset of COI, 28S and ALG11 for a selection of cladorhizids of the genera *Asbestopluma*, *Abyssocladia*, *Chondrocladia*, and *Cladorhiza*, and **(B)** Polymastiidae based on a concatenated dataset of COI and 28S. E2 *Cladorhiza* and Endeavour *Spinularia* sp. specimens examined in the present study are indicated by white circles, while specimens known to have been collected near vent or seep environments are highlighted in blue. For a list of the GenBank accession numbers for the sequences used, please refer to Supplementary Tables S2, S3.

Phylogenetic analysis for Cladorhizidae recovered two subclades within *Cladorhiza* with high support (Figure 3A), one comprising *C. kenchingtonae* and *Cladorhiza* sp. SMF11751 and the other comprising all other *Cladorhiza* species included in the analysis. Despite E2 *Cladorhiza* specimens in our study being collected at the same site (Figure 1A), and not showing notable differences in their spicules (Eck and Janussen, Unpublished), they appeared to be two different lineages. Three of our specimens (F-0135A, F-0135B, and PS119-46) formed a clade, *Cladorhiza* sp. A, closely related to the species *C. methanophila* and *C. gelida*, described from seeps of the Barbados Trench at approximately 4700 m depth (Vacelet and Boury-Esnault, 2002), and from between Jan Mayen and Iceland in the Norwegian sea at 1100–2300 m depth (Lundbeck, 1905), respectively. An additional E2 *Cladorhiza* individual (F-0146; *Cladorhiza* sp. B) instead showed close affinities to a specimen of the north-east Atlantic species *C. abyssicola*, collected from the Skagerrak (Hestetun et al., 2016b). Genetic distances between the two E2 *Cladorhiza* lineages were greater than 2 and 4% for COI and ALG11, respectively (and a maximum of 0.6 and 0% between E2 *Cladorhiza* sp. A specimens F-0135A, F-0135B, and PS119-46; Supplementary Tables S5–S7). Figure 3A also indicates relationships between a subset of the *Chondrocladia* species used as part of the microbiome analysis in this study, with *C. grandis* and *C. robertballardi* falling within one clade that forms a sister group to a clade comprised of the species *C. verticillata* and *Chondrocladia* sp. from Patagonia.

The 11 *Spinularia* sp. specimens from Endeavour formed a single clade (Figure 3B). They demonstrated well-supported close relationships to the species *Spinularia njordi*, *Spinularia spinularia*, *Spinularia sarsii*, and *Spinularia* cf. *sarsii*, collected from the North and Norwegian Seas (Plotkin et al., 2017, 2018), with genetic distances between the above species and Polymastiidae from Endeavour being less than 2% for COI and 0.8% for 28S. Genetic distances between *Spinularia* species and other polymastiids were greater than 3.3% for COI and 1.7% for 28S (Supplementary Tables S8, S9).

### Isotopic Analyses

Stable carbon isotope ratios of the sponges were depleted in ^13^C and ranged from −27.0‰ in the E2 *Cladorhiza* sp. A specimen (PS119-46; Table 1), to −33.6 and −33.4‰ in the two Endeavour *Spinularia* sp. specimens (028-S2.1 and 034-S2.3, respectively, Table 1). Similarly, sponge stable nitrogen isotope ratios were depleted in ^15^N and ranged from 4.2‰ in the E2 *Cladorhiza* sp. A specimen, to 1.3 and 2.3‰ in the two Endeavour *Spinularia* sp. specimens.

### Microbiome Composition

Microbiome sequencing resulted in 16S rRNA amplicon reads for 25 samples representing 19 sponge individuals (Table 1 and Supplementary Table S4) spread across the two datasets, *Cladorhiza-Chondrocladia* (Dataset 1) and *Spinularia-Sycon* (Dataset 2). Total filtered reads for each sample varied between 29,836–78,379 (79% of initial reads; Dataset 1) and 75,180–208,797 (71% of initial reads; Dataset 2) (Supplementary Table S4). Final total numbers of unique ASVs for each dataset were 6,058 and 20,067, respectively. For microbiome data generated by Hestetun et al. (2016a) (Dataset 3; Supplementary Table S4), application of the processing steps above filtered 31% of reads, resulting in 7,873 unique ASVs for the dataset and total reads for each sample varying between 35,801 and 233,944.

**FIGURE 4:**
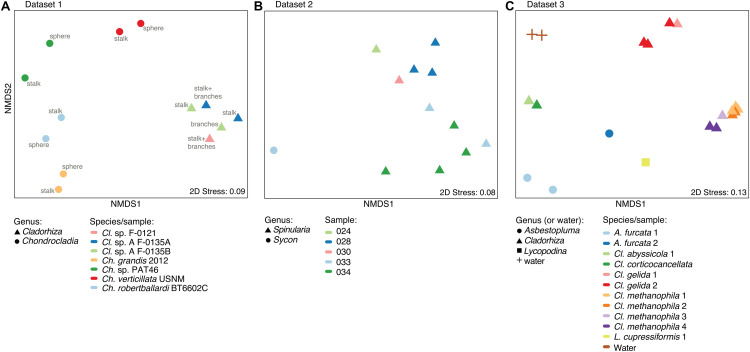
Microbiome similarity in sponge samples as determined by non-metric multi-dimensional scaling (nMDS) ordination analysis. The plots illustrate nMDS results for the two microbiome datasets generated during this study and the additional dataset of Hestetun et al. (2016a), while points are shaped according to taxonomic affinity (genus) and colored by species/sampling event. **(A)** nMDS plot for Dataset 1, *Cladorhiza-Chondrocladia*. **(B)** nMDS plot for Dataset 2, *Spinularia-Sycon*. **(C)** nMDS plot for Dataset 3 generated by Hestetun et al. (2016a).

**FIGURE 5:**
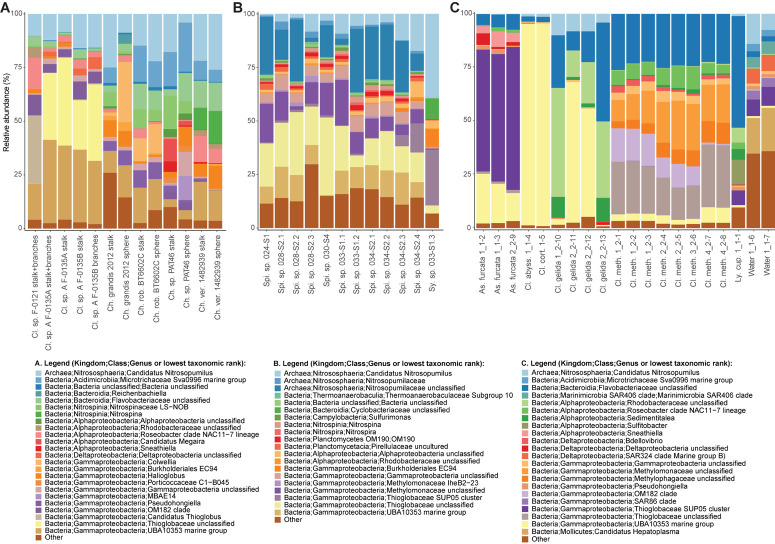
Relative abundances of the most common prokaryotes for individual sponge samples at the genus level. Genera with relative abundances lower than 0.5% across the dataset were pooled into the category “Other”. The bar charts illustrate microbiome composition for the two microbiome datasets generated during this study, **(A)** *Cladorhiza-Chondrocladia* (Dataset 1) and **(B)** *Spinularia-Sycon* (Dataset 2), while **(C)** illustrates the above for the Cladorhizidae dataset generated by Hestetun et al. (2016a) (Dataset 3). Genus and species names are abbreviated as follows: Cl. *Cladorhiza*, Ch. *Chondrocladia*, rob. *robertballardi*, ver. *verticillata*, Ly. cup. *Lycopina cupressiformis*, As. *Asbestopluma*, meth. *methanophila*, abyss. *abyssicola*, cort. *corticocancellata*, Spi. *Spinularia*, Sy. *Sycon*.

A nMDS plot generated for the *Cladorhiza-Chondrocladia* dataset (Dataset 1) showed grouping by genus (Figure 4A). PERMANOVA tests confirmed a significant difference between *Cladorhiza* and *Chondrocladia* microbiomes (*p* value = 0.002), and no difference between the tissue types analyzed (i.e., spheres, stalk, branches or stalk+branches; *p* value = 0.49). Upon taxonomic assignment of the ASVs, this dataset contained 45 bacterial and 5 archaeal phyla, with Proteobacteria and Thaumarchaeota being the most abundant phyla, while Gammaproteobacteria were the dominant microbial class (Supplementary Table S10). At lower taxonomic ranks, proportions of the main microbial taxa noticeably differed between E2 *Cladorhiza* specimens and *Chondrocladia* spp. (Figure 5A and Supplementary Figure S1A). The majority of Gammaproteobacteria in the dataset belonged to the UBA10353 marine group and to Thioglobaceae, with both of these gammaproteobacterial taxa being most abundant within E2 *Cladorhiza* specimens. Thioglobaceae was only observed within E2 *Cladorhiza* (Figure 5A and Supplementary Table S10). There appeared to also be a degree of variation in microbiomes between different E2 *Cladorhiza* specimens, as one of the samples (F-0121) contained a different dominant Thioglobaceae genus and a greater proportion of Alphaproteobacteria than the others (Figure 5A and Supplementary Table S10), however variations between different tissue types remained small. NCBI GenBank BLAST searches of the two dominant Thioglobaceae lineages observed in E2 *Cladorhiza* specimens were highly similar (98% similarity) to *Bathymodiolus* thiotrophic gill symbionts (GenBank accessions: CP024634.1, LN871183.2, KF521929.1, AP013042.1, and KF521926.1) as well as to uncultured bacteria. *Chondrocladia* spp. were instead generally dominated by *Nitrosopumilus* archaea, which also accounted for 8–16% of reads in E2 *Cladorhiza* specimens (Supplementary Table S10) and were found to have 100% similarity to an uncultured archaeon (GenBank accession AB327651). In addition, *Chondrocladia* spp. showed high proportions of Acidimicrobiia, especially the Microtrichales group Sva0996, and Nitrospinia, which are mostly nitrite oxidizers.

In comparison, microbiome reads for cladorhizids generated by Hestetun et al. (2016a) (Dataset 3) showed species-specific microbial assemblages (Figure 4C; PERMANOVA *p* value = 0.001), which were also significantly different between the methanotrophic species *Cladorhiza methanophila* and the other species (*p* value = 0.001). Microbiomes in this dataset were generally dominated by the bacterial phyla Proteobacteria and Bacteroidetes, while Thaumarchaeota was only observed within the species *Cladorhiza gelida* (Supplementary Table S12). Class and genus-level results reflected those of previous analyses (Hestetun et al., 2016a), and demonstrated notable differences in the occurrence of Methylomonaceae [reported as Methylococcales in Hestetun et al. (2016a)], Thioglobaceae and OM182 clade Gammaproteobacteria, which were abundant within *C. methanophila* specimens but not in other cladorhizids (Figure 5C, Supplementary Figure S1C and Supplementary Table S12). GenBank BLAST searches of *C. methanophila* Thioglobaceae also showed high similarity to uncultured bacteria as well as to *Bathymodiolus* thiotrophic gill symbionts (98% similarity; CP024634.1, LN871183.2). There was only one base-pair difference between the sequences of *C. methanophila* Thioglobaceae and the dominant E2 *Cladorhiza* Thioglobaceae. *C. methanophila* Methylomonaceae was similar to seep and mud volcano bacteria (99% similarity to GenBank accessions JN884825.1 and FJ712568.1).

The *Spinularia-Sycon* dataset (Dataset 2) also indicated that species-specificity is the dominant factor separating samples. However, there also appeared to be some differences between Endeavour *Spinularia* sp. collection locations (Figure 4B). Dataset 2 contained 40 bacterial phyla of which Proteobacteria was the most abundant followed by the archaeal phylum Thaumarchaeota, while at the class level, Gammaproteobacteria dominated the dataset followed by archaea of the class Nitrososphaeria (Supplementary Table S11). *Spinularia* specimens were dominated by the Gammaproteobacteria Thioglobaceae, UBA10353, and Methylomonaceae, as well as archaea of the family Nitrosopumilaceae (Figure 5B, Supplementary Figure S1B and Supplementary Table S11). Proportions of microbe genera were somewhat variable between different *Spinularia* individuals (Figure 5B), which usually contained large proportions of low-abundance microbes (genera with less than 0.5% relative abundance across each dataset are grouped under the category “Other”), especially in one individual where they comprised nearly 28% of the microbiome (Figure 5B, individual Spi. sp. 028-S2.3). In contrast, the microbiome of *Sycon* sp. (Sy. sp. 033-S1-3) collected near to two *Spinularia* specimens (Spi. sp. 033-S1.1, Spi. sp. 033-S1.2) contained high proportions of Candidatus *Nitrosopumilus* archaea, as well as of SUP05 cluster Gammaproteobacteria (Figure 5B and Supplementary Figure S1B).

When searched against existing microbial sequences available on GenBank, the *Spinularia* sp. Thioglobaceae were very similar (98% similarity) to *Bathymodiolus* gill symbionts (e.g., GenBank accessions: AB499797.1, KF657323.1), while Methylomonaceae were similar to seep and mud volcano Gammaproteobacteria (98% similarity to GenBank accessions JN884825.1 and FJ712568.1). There were 10 base-pair differences between E2 *Cladorhiza* and *Spinularia* sp. Thioglobaceae, and four base-pair differences between *Cladorhiza methanophila* and *Spinularia* sp. Methylomonaceae. For archaea, those dominating the *Sycon* sp. sample were 100% match to *Nitrosopumilus* within Dataset 1, while dominant archaea within *Spinularia* sample 024-S1 showed a maximum of 98.4% similarity to existing GenBank accessions such as MK139956.

## Discussion

This study comprises the first attempt to explore the nutritional sources, microbiomes, and evolutionary histories of vent-peripheral sponges, from different vent environments and with distinct inferred feeding modes, to provide an indication of the ways in which sessile metazoans living on the edge of vent zones benefit from these highly productive deep-sea environments. Our results suggest that the vent peripheral sponges examined here potentially obtain part of their nutrition from vent-driven chemosynthesis, carried out by gammaproteobacterial lineages specific to known chemosynthetic species, which were found to be present in high abundances in the contrasting sponge taxa that we examined.

### Evolutionary Histories of Vent-Peripheral *Cladorhiza* and *Spinularia* sp.

Our phylogenetic results indicate an evolutionary association with chemosynthetic environments for one of the vent-peripheral sponges analyzed in this study, as a close, well-supported sister relationship was uncovered between three E2 *Cladorhiza* sp. A individuals and the known methanotrophic seep-dwelling species *Cladorhiza methanophila* (Figure 3A). *C. methanophila* is considered an isolated case in this genus of a dependence on chemolithoautotrophic symbiosis (Hestetun et al., 2016a). However, the very high similarity in Thioglobaceae lineages between *C. methanophila* and E2 *Cladorhiza* sp. A may indicate an adaptive association with these bacteria to life within chemosynthetic environments. The genus *Cladorhiza* appears to be represented at E2 by at least two species with quite different descent (Figure 3A), as one of the E2 *Cladorhiza* individuals (*Cladorhiza* sp. B) is instead most-closely related to the North Atlantic species *C. abyssicola*. This relationship highlights another evolutionary link between high-latitude Atlantic deep-sea ecosystems, as recently demonstrated by the bipolar vent/seep annelid species *Nicomache lokii* and *Sclerolinum contortum* (Georgieva et al., 2015; Eilertsen et al., 2018). Whether the two distinct E2 *Cladorhiza* species exhibit ecological niche overlap at this vent site is at present unclear, as we were not able to acquire microbiome or isotopic data for E2 *Cladorhiza* sp. B specimen F-0146. We were also unable to sequence barcoding genes for E2 *Cladorhiza* specimen F-0121, which contained a different Thioglobaceae to other individuals (Figure 5A). This difference may therefore be due to a difference between species, or potentially a case of symbiont optimization, as reported for *Bathymodiolus* mussels (Ansorge et al., 2019).

In contrast, all collected individuals of *Spinularia* sp. from Endeavour belong to the same species (Figure 3B). Only four species of *Spinularia* are currently known mostly from the northern Atlantic, with the Endeavour *Spinularia* sp. specimens appearing most closely related to *S. njordi*, described from near the Loki’s Castle vents on the Mid-Atlantic Ridge, however, with low support. None are currently considered to be able to feed through chemosynthesis. The Endeavour *Spinularia* sp. specimens represent a new north-east Pacific species of this little-studied genus.

### Indications of Nutritional Sources for Vent-Peripheral Sponges

Isotopic analyses of E2 *Cladorhiza* sp. A and Endeavour *Spinularia* sp., while limited in number, showed that these sponges were depleted in both ^13^C and ^15^N (Supplementary Figure S2). The ^13^C-depleted values of the sponges in this study likely indicate the assimilation of carbon fixation fuelled by energy derived from sulfide oxidation via the Calvin-Benson-Bassham (CBB) cycle utilizing form I RuBisCO, if ambient dissolved inorganic carbon was used as a carbon source. Our δ^13^C results are in close agreement with previous measurements for E2 *Cladorhiza* by Reid et al. (2013), who suggest that at the East Scotia Ridge hydrothermal vents, δ^13^C values of less than −22‰ are indicative of CBB cycle-assimilated carbon. At Endeavour, δ13C values of −29 to −33‰ have been reported in a range of taxa considered to be reliant on chemosynthetic food sources, that are either associated with chemosynthetic symbionts or graze on microbial mats (Bergquist et al., 2007). Our carbon isotope data therefore points to a potential nutritional association with chemosynthetic bacteria (Bergquist et al., 2007; Reid et al., 2013).

The depleted δ^15^N values obtained for sponges in this study are lower than previous results for *Cladorhiza* from E2 (Reid et al., 2013), being closer to those reported for *C. methanophila* from mud-volcanoes in the Barbados Accretionary Prism (Hestetun et al., 2016b). These values indicate fixation and/or assimilation of local inorganic nitrogen, which could result from nitrogen fixation by diazotrophs (Conway et al., 1994; Levin and Michener, 2002). Isotopically depleted δ^15^N values can also result from uptake of nitrate and/or ammonium depleted in ^15^N by symbionts (Fisher, 1995), or from the activity of sulfur-oxidizing bacteria, as reported from cold seep environments (Levin and Michener, 2002; Demopoulos et al., 2010; Decker and Olu, 2012).

### The Microbiomes of Vent-Peripheral Sponges

Our exploration of the microbiomes of the vent-peripheral sponges *Cladorhiza* from E2 and *Spinularia* sp. from Endeavour surprisingly indicated high proportions of Gammaproteobacteria, in particular Thioglobaceae within E2 *Cladorhiza* specimens, and Thioglobaceae and Methylomonaceae within Endeavour *Spinularia* sp. Thioglobaceae accounted for 23–40% of the microbiome of E2 *Cladorhiza* specimens, while Thioglobaceae and Methylomonaceae accounted for 15–51% of *Spinularia* sp. microbiomes (Supplementary Tables S10, S11). These microbes are unlikely to have been captured from the water column by the sponges, as comparisons between cladorhizid microbiomes and those of water samples collected nearby indicate clear differences in the relative proportions of gammaproteobacterial groups (Hestetun et al., 2016a; Figure 5C).

Thioglobaceae and Methylomonaceae also do not occur in the non-vent *Chondrocladia* specimens. Instead, the Gammaproteobacteria detected in vent-peripheral *Cladorhiza* and *Spinularia* sp. sponges are highly similar to gammaproteobacterial endosymbionts of chemosynthetic mussels *Bathymodiolus* sp. (Fontanez and Cavanaugh, 2014; Sayavedra et al., 2015; Ikuta et al., 2016) as well as of the sponge *C. methanophila* (Hestetun et al., 2016a). This study therefore adds to a growing number of observations of Gammaproteobacteria belonging to the Thioglobaceae and Methylomonaceae lineages occurring in association with diverse metazoan hosts at chemosynthetic environments (Rubin-Blum et al., 2019; Zhou et al., 2019; Goffredi et al., 2020; Vohsen et al., 2020), and provides the first indication that such associations also occur within the peripheral areas of hydrothermal vent environments, away from obvious flow of reduced fluids from the seafloor.

Whilst further in-depth study would be needed to elucidate the relationships between Thioglobaceae bacteria and the vent peripheral sponges *Cladorhiza* and *Spinularia* sp., as well as the relationship between Methylomonaceae and *Spinularia* sp., Gammaproteobacteria are in general the main sulfide-oxidizing symbionts of vent and seep fauna (Dubilier et al., 2008), and are also the most important symbionts of sponges with known chemosynthetic symbioses (Hestetun et al., 2016a; Rubin-Blum et al., 2019; Zhou et al., 2019). In particular, the Thioglobaceae and Methylomonaceae observed within the sponges examined in this study are also known to associate with other sponges from deep-sea chemosynthetic environments, where they perform sulfide and/or methane oxidation (Rubin-Blum et al., 2019; Zhou et al., 2019). The similarity between the Thioglobaceae in the sponges analyzed here and *Bathymodiolus* thiotrophic gill symbionts also indicates that the Thioglobaceae found within E2 *Cladorhiza* specimens and Endeavour *Spinularia* sp. are likely capable of sulfide oxidation, and may be responsible for the observed depleted δ^13^C values.

It is also interesting to note that while the majority of E2 *Cladorhiza* individuals analyzed here (*Cladorhiza* sp. A) show a close evolutionary relationship to the known chemosynthesis-dependent species *Cladorhiza methanophila* (Figure 3A) and contain very similar Thioglobaceae lineages, they also exhibit differences in their microbial associates, with *C. methanophila* containing high proportions of methane-oxidizing Methylomonaceae and Methylophagaceae that are absent in E2 *Cladorhiza* sp. A (Figure 5A). This suggests surprising flexibility in microbial associate acquisition between these closely-related sponges, which reflects findings for *Bathymodiolus* mussels that are capable of associating with either sulfide oxidizing symbionts, methane oxidizing symbionts, or both (Duperron et al., 2009; Szafranski et al., 2015).

Gammaproteobacterial groups other than Thioglobaceae and Methylomonaceae also appear to be important associates of vent-peripheral sponges. For vent-peripheral cladorhizids deemed to be partially reliant but not dependent on chemosynthetic symbionts for nutrition (Hestetun et al., 2016a), the UBA10353 marine group Gammaproteobacteria occur in high proportions in the species *Asbestopluma furcata*, *C. abyssicola*, *C. corticocancellata* and two *C. gelida* samples, while SUP05 Thioglobaceae dominates *A. furcata* samples (Figure 5 and Supplementary Table S12). The UBA10353 marine group were also found to comprise 17–39% of E2 *Cladorhiza* and 0–15% of Endeavour *Spinularia* microbiomes (Figure 5 and Supplementary Tables S10, S11). However, information on their distribution and function is limited because many of these microbes have not been cultured and have not yet been widely detected in the environment. UBA10353 Gammaproteobacteria closely related to those observed in E2 and Endeavour vent-peripheral sponges have also been observed at seeps (GenBank accession MH885636 98.8% similar to E2 *Cladorhiza* samples) as well as in non-chemosynthetic environments (GenBank accession KF597133 98% similar to Endeavour *Spinularia* sp. UBA10353 sequences, Kennedy et al. (2014)). This suggests that they can occur in a range of marine settings. SUP05 cluster Gammaprotobacteria dominant in *A. furcata* samples as well as the Endeavour *Sycon* sp. individual are known chemolithoautotrophic symbionts of Haplosclerida sponges from vents (Zhou et al., 2019), but are also generally abundant and ubiquitous in various marine environments (Walsh et al., 2009; Anantharaman et al., 2013, 2014).

The present study has also uncovered the dominance of *Nitrosopumilus* archaea within deep-sea sponges, which made up significant proportions of microbiomes in both Datasets 1 and 2 (Figures 5A,B). These are considered to be ammonia-oxidizing but can have diverse functions (Bayer et al., 2016), and this study has also revealed novel types within Endeavour *Spinularia* sp. sponges.

For both E2 *Cladorhiza* and Endeavour *Spinularia* sp. sponges, the above microbial associations occur in a setting that is not subjected to elevated temperatures and has no visible fluid flow. Additionally, there are no indications of reduced chemicals in the form of organisms typical of low flow vent settings, such as microbial mats on basalt surfaces. But the types of Gammaproteobacteria found within the examined vent-peripheral sponges do indicate a direct relation to nearby vents, due to their similarity to chemosynthetic symbionts of animals inhabiting vent and seep environments. In the absence of signs of fluid emission in the vent periphery, a possible way in which reduced chemicals from the vents may be reaching vent-peripheral sponges is through lateral dispersal of vent effluent. The presence of pyrite grains observed on the branches of an E2 *Cladorhiza* sp. A specimen (Figure 2B) indicate that vent fluids do reach these sponges.

At Endeavour, concentrations of methane within the vent fluid are unexpectedly high (Butterfield et al., 1994), which may account for why *Spinularia* sp. sponges collected at this site also contain high proportions of potentially methane-oxidizing Methylomonaceae. Relative abundances of Thioglobaceae and Methylomonaceae within *Spinularia* sp. individuals do not appear correlated to distances from vent chimneys (Figures 1A, 5B). However, local current circulation (Thomson et al., 2009) may be of greater importance. Given that sulfide oxidation is one of the main microbial functions occurring within the vent plume (Anantharaman et al., 2016), it is highly likely that some of these sulfide oxidizers may associate with animals in the vent periphery from which they may be able to obtain more stable and optimal access to the vent plume chemicals. In addition, the high filtration rates of some sponges (Kahn et al., 2015) may enable them to concentrate chemicals from the vent plume, enabling chemosynthesis to occur at a considerable distance from vents. Mapping the distributions of *Cladorhiza* and *Spinularia* sp. in relation to vents and local currents at their respective vent sites would help to elucidate the importance of the plume to these sponges.

## Conclusion

By investigating the microbiomes of two distantly-related vent-peripheral sponges, as well as examining their evolutionary history and nutritional sources, our results have detected a close sister relationship between a subset of *Cladorhiza* specimens from the E2 hydrothermal vents and the known chemosynthesis-reliant seep sponge *Cladorhiza methanophila*. We have also uncovered the presence of potentially chemosynthetic Gammaproteobacteria within both *Cladorhiza* and *Spinularia* sp. sponges living at the periphery of hydrothermal vent fields. The above associations are consistent with isotopic data obtained for these sponges. The similarity of the Gammaproteobacteria found within the vent-peripheral sponges examine here to microbes reported in other vent and/or seep dwelling species suggest that chemosynthesis may also be occurring at the vent periphery within these sponges, possibly driven by the lateral dispersal of vent fluids away from the main vicinity of chimneys, and that they are thus adapted to the vent-peripheral habitat they occupy. It is plausible that sponges, with their adaptations to high-volume water filtration (Kahn et al., 2015), have a unique ability to obtain chemosynthetic food sources in the vent periphery. The suggestion of these findings of vent-driven chemosynthetic symbioses existing in the vent periphery reflects recent findings for deep-sea chemosynthetic environments in general, where nutrient supplies to the surrounding deep sea are increasingly found to be more widespread than originally anticipated (Levin et al., 2016; Seabrook et al., 2019; Goffredi et al., 2020; Vohsen et al., 2020). Our findings indicate that the vent periphery maybe an important and overlooked biotic zone of deep-sea hydrothermal vents with significant local ecological effects as well as a driver of evolutionary novelty in the deep sea.

## Data Availability Statement

The datasets presented in this study can be found in online repositories. The names of the repository/repositories and accession number(s) can be found at: https://www.ncbi.nlm.nih.gov/, BioProject accession number PRJNA635099, sample accession numbers SAMN15016193 to SAMN15016217 https://www.ncbi.nlm.nih.gov/genbank/, accession numbers MT521886 to MT521916.

## Author Contributions

MG, AR, AG, and JC conceived of the study. JC, FD, CL, PR, JC, and JH collected the specimens. MG, ST and CD performed the molecular lab work and data analysis. RJ conducted isotopic analyses. MG led the original draft preparation. All authors contributed to the writing-review and editing of the final version of the manuscript.

## Conflict of Interest

The authors declare that the research was conducted in the absence of any commercial or financial relationships that could be construed as a potential conflict of interest.

## Supplementary Material

FIGURE S1 Relative abundances (log10 transformed) of the most common prokaryotes for individual sponge samples at the genus level. Genera with relative abundances lower than 0.5% across the dataset were omitted from the figure. The heatmaps also illustrate similarity (weighted UniFrac distance cladograms) of microbiome composition for each microbiome dataset analyzed, **(A)** *Cladorhiza-Chondrocladia* (Dataset 1) and **(B)** *Spinularia-Sycon* (Dataset 2), while **(C)** Cladorhizidae dataset generated by Hestetun et al. (2016a) (Dataset 3). Genus and species names are abbreviated as follows: Cl. *Cladorhiza*, Ch. *Chondrocladia*, rob. *robertballardi*, ver. *verticillata*, Ly. cup. *Lycopina cupressiformis*, As. *Asbestopluma*, meth. *methanophila*, abyss. *abyssicola*, cort. *corticocancellata*, Spi. *Spinularia*, Sy. *Sycon*.

FIGURE S2 Carbon and nitrogen isotopic results (δ^13^C and δ^15^N) for E2 *Cladorhiza* and Endeavour *Spinularia* sp. specimens analyzed during the present study in comparison to results for cladorhizid sponges from the previous studies of Erickson et al. (2009), Reid et al. (2013), and Hestetun et al. (2016a).

TABLE S1 Primers used for amplification and sequencing of DNA fragments used in phylogenetic analyses.

TABLE S2 Genbank sequence codes of specimens used in phylogenetic analyses of Cladorhizidae.

TABLE S3 Genbank sequence codes of specimens used in phylogenetic analyses of Polymastiidae.

TABLE S4 Overview of the datasets used for 16S rRNA sequencing.

TABLE S5 COI genetic distances (*p*-distance) for the genus *Cladorhiza*.

TABLE S6 28S genetic distances (*p*-distance) for the genus *Cladorhiza*.

TABLE S7 ALG11 genetic distances (*p*-distance) for the genus *Cladorhiza*.

TABLE S8 COI genetic distances (*p*-distance) for the family Polymastiidae.

TABLE S9 28S genetic distances (*p*-distance) for the family Polymastiidae.

TABLE S10 Relative abundance of the 10 most common prokaryotes over the *Cladorhiza-Chondrocladia* dataset (Dataset 1) at the phylum, class and genus level. Values over 5% are highlighted.

TABLE S11 Relative abundance of the 10 most common prokaryotes over the *Spinularia-Sycon* dataset (Dataset 2) at the phylum, class and genus level. Values over 5% are highlighted.

TABLE S12 Relative abundance of the 10 most common prokaryotes over the Hestetun et al. (2016a) dataset (Dataset 3) at the phylum, class and genus level. Values over 5% are highlighted.
