# Supplementary material for: Evidence of Vent-Adaptation in Sponges Living at the Periphery of Hydrothermal Vent Environments: Ecological and Evolutionary Implications
Source: Front Microbiol. 2020 Jul 24;11:1636. doi: 10.3389/fmicb.2020.01636 (PMC7393317; doi:10.3389/fmicb.2020.01636)
Supplement: FIGURE S1 — Relative abundances (log10 transformed) of the most common prokaryotes for individual sponge samples at the genus level. Genera with relative abundances lower than 0.5% across the dataset were omitted from the figure. The heatmaps also illustrate similarity (weighted UniFrac distance cladograms) of microbiome composition for each microbiome dataset analyzed, (A) Cladorhiza-Chondrocladia (Dataset 1) and (B) Spinularia-Sycon (Dataset 2), while (C) Cladorhizidae dataset generated by Hestetun et al. (2016a) (Dataset 3). Genus and species names are abbreviated as follows: Cl. Cladorhiza, Ch. Chondrocladia, rob. robertballardi, ver. verticillata, Ly. cup. Lycopina cupressiformis, As. Asbestopluma, meth. methanophila, abyss. abyssicola, cort. corticocancellata, Spi. Spinularia, Sy. Sycon. [file Data_Sheet_1.pdf]

Supplementary figures to:

Georgieva MN, Taboada S, Riesgo A, Díez-Vives C, De Leo FC, Jeffreys RM, Copley JT, Little CTS, Ríos P, Cristobo J, Hestetun JT and Glover AG (2020) Evidence of vent-adaptation in sponges living at the periphery of hydrothermal vent environments: ecological and evolutionary implications. Frontiers in Microbiology 11:1636. doi: 10.3389/fmicb.2020.01636

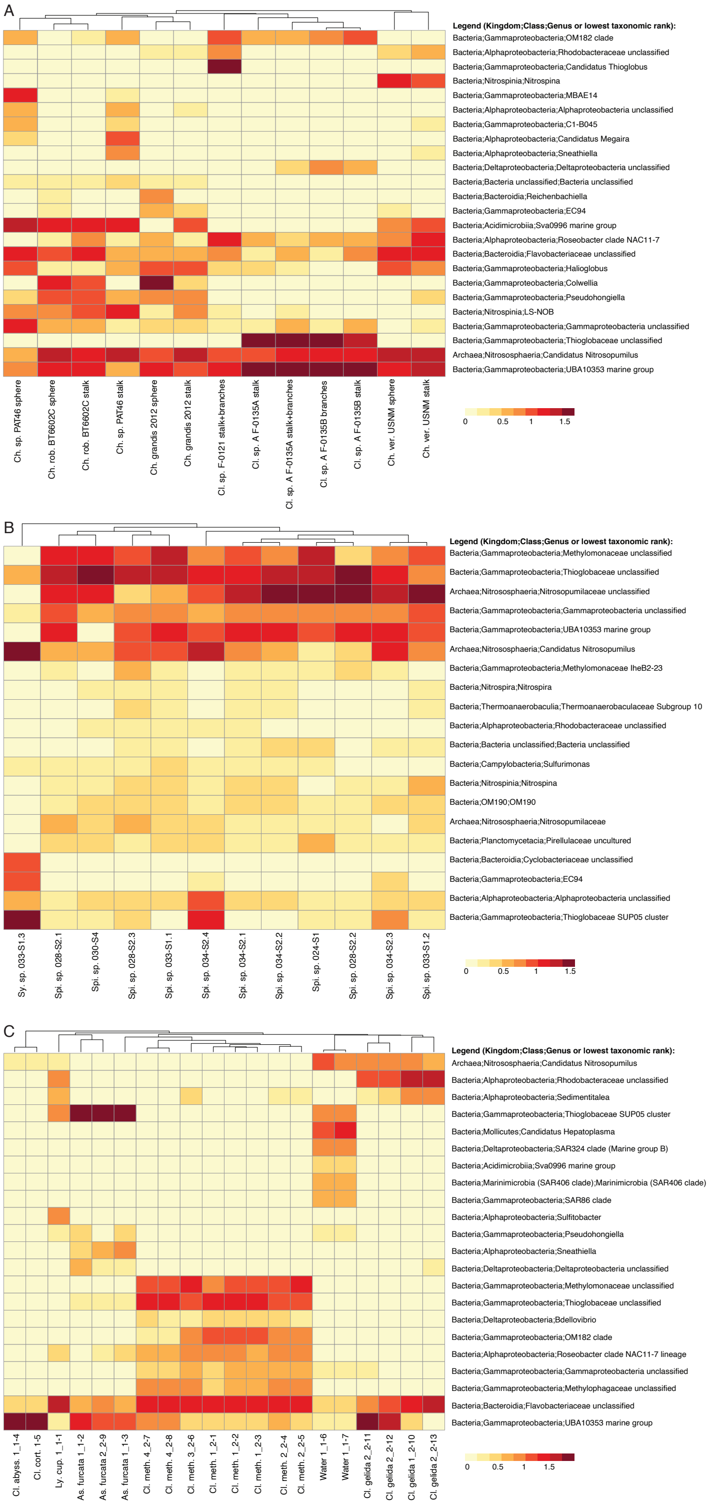

Figure S1. Relative abundances (log10 transformed) of the most common prokaryotes for individual sponge samples at the genus level. Genera with relative abundances lower than 0.5% across the dataset were omitted from the figure. The heatmaps also illustrate similarity (weighted UniFrac distance cladograms) of microbiome composition for each microbiome dataset analysed, **A,** *Cladorhiza-Chondrocladia* (Dataset 1) and **B**, *Spinularia-Sycon* (Dataset 2), while **C**, Cladorhizidae dataset generated by Hestetun et al. (2016) (Dataset 3). Genus and species names are abbreviated as follows: Cl. *Cladorhiza*, Ch. *Chondrocladia*, rob. *robertballardi*, ver. *verticillata*, Ly. cup. *Lycopina cupressiformis*, As. *Asbestopluma*, meth. *methanophila*, abyss. *abyssicola*, cort. *corticocancellata*, Spi. *Spinularia*, Sy. *Sycon*.

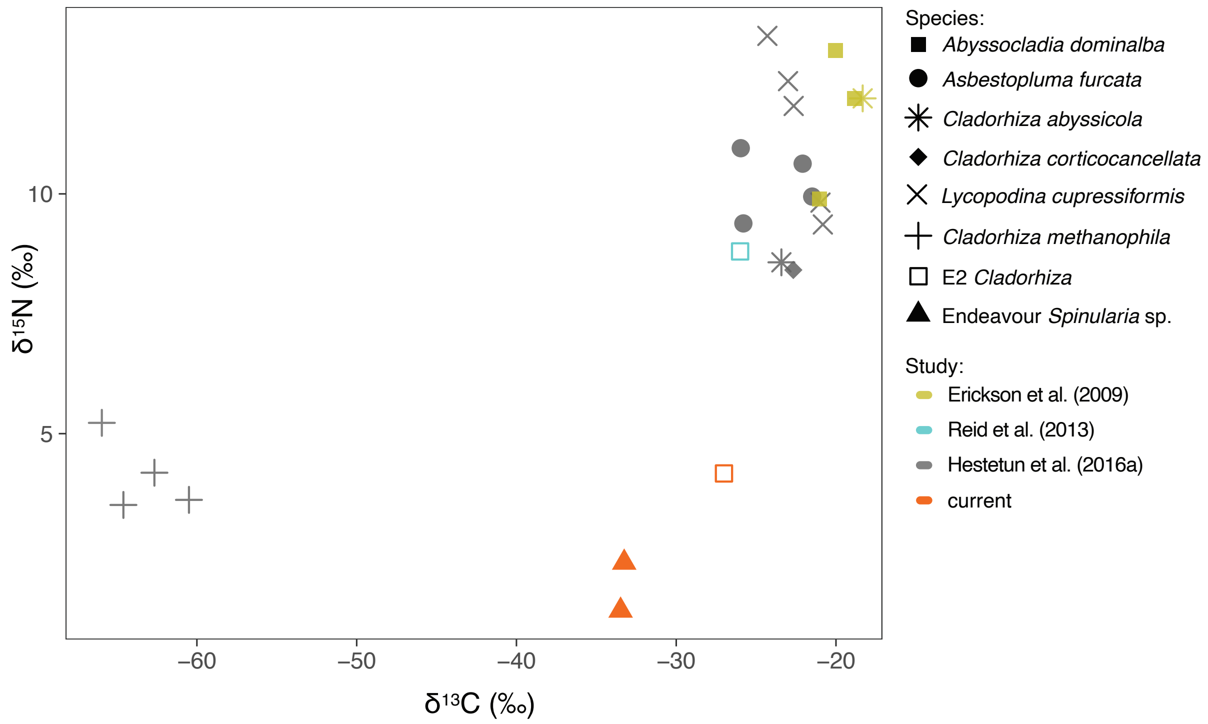

Figure S2. Carbon and nitrogen isotopic results ($\delta^{13}C$ and $\delta^{15}N$) for E2 *Cladorhiza* and Endeavour *Spinularia* sp. specimens analysed during the present study in comparison to results for cladorhizid sponges from the previous studies of Erickson et al., (2009), Reid et al. (2013), Hestetun et al. (2016).

References

Erickson, K. L., Macko, S. A., and Van Dover, C. L. (2009). Evidence for a chemoautotrophically based food web at inactive hydrothermal vents (Manus Basin). *Deep Sea Res. Part II Top. Stud. Oceanogr.* 56, 1577–1585. doi:10.1016/j.dsr2.2009.05.002.

Hestetun, J. T., Dahle, H., Jørgensen, S. L., Olsen, B. R., and Rapp, H. T. (2016). The microbiome and occurrence of methanotrophy in carnivorous sponges. *Front. Microbiol.* 7, 1781. doi:10.3389/fmicb.2016.01781.

Reid, W. D. K., Sweeting, C. J., Wigham, B. D., Zwirglmaier, K., Hawkes, J. A., McGill, R. A. R., et al. (2013). Spatial differences in East Scotia Ridge hydrothermal vent food webs: influences of chemistry, microbiology and predation on trophodynamics. *PLoS One* 8, e65553. doi:10.1371/journal.pone.0065553.
